# Effects of a Nutrigenetic Diet on Fat Mass Loss, Insulin Resistance, and Subclinical Inflammation in Adults with Abdominal Obesity: A Randomized Controlled Trial—The INNUPREC Study

**DOI:** 10.3390/nu18213519

**Published:** 2026-10-26

**Authors:** Alondra Mora-Jiménez, Wendy Campos-Perez, Erika Martinez-Lopez

**Affiliations:** 1 Universidad de Guadalajara, Centro Universitario de Ciencias de la Salud, Departamento de Biología Molecular y Genómica, Doctorado en Ciencias en Biología Molecular en Medicina, Guadalajara 44340, Jalisco, Mexico; alondra.mora2239@alumnos.udg.mx; 2 Universidad de Guadalajara, Centro Universitario de Ciencias de la Salud, Departamento de Biología Molecular y Genómica, Instituto de Nutrigenética y Nutrigenómica Traslacional, Guadalajara 44340, Jalisco, Mexico; wendy.campos4381@academicos.udg.mx

**Keywords:** nutrigenetic diet, abdominal obesity, genetic variants, precision nutrition

## Abstract

**Background:** Abdominal obesity (AO) is involved in the development of cardiometabolic disorders, in which factors such as diet and genetics play crucial roles. In this context, interactions between genetic variants and dietary responses may influence metabolic processes associated with AO. **Objective**: This study aimed to evaluate the effects of a nutrigenetic diet incorporating 21 genetic variants related to lipid metabolism and cardiovascular risk on anthropometric and biochemical parameters and antioxidant capacity in adults with AO. **Methods**: An eight-week randomized controlled trial was conducted in 58 adults with AO, who were randomly assigned to either a nutrigenetics-guided diet or a standard diet. Participants in the nutrigenetic group underwent genotyping for 21 variants associated with lipid metabolism and cardiometabolic risk in the *ABCA1*, *APOA1*, *APOA2*, *APOA5*, *APOB*, *APOC3*, *APOE*, *CETP*, *CDKN2B-AS1*, *CD36*, *FABP2*, *FTO*, *LIPC*, *LPL*, *MTTP*, *MTHFR*, *NFKB1*, *PON1*, *SIRT1*, and *ZPR1* genes. Anthropometric and biochemical measurements were performed. Antioxidant and oxidative stress biomarkers were measured using colorimetric assays and ELISA, respectively. **Results:** In baseline-adjusted ANCOVA models controlling for age and sex, with false discovery rate (FDR) correction, both groups achieved comparable reductions in body weight and fat mass under equivalent energy restriction. However, the nutrigenetic group demonstrated significantly greater improvements in insulin levels, HOMA-IR, and high-sensitivity *C*-reactive protein (hs-CRP) compared with the standard diet group. The nutrigenetic intervention also resulted in greater reductions in the prevalence of elevated TyG index (−20% vs. −10.8%) and HOMA-IR (−33.3% vs. −10.7%), along with a trend toward a greater increase in catalase activity. **Conclusions**: Compared with a standard dietary intervention, a nutrigenetic diet was associated with greater improvements in fasting insulin, estimated insulin resistance (HOMA-IR), and systemic inflammation (hs-CRP) in adults with AO.

## 1. Introduction

Cardiovascular diseases (CVDs) are a leading cause of morbidity and mortality among adults worldwide. Abdominal obesity (AO) is a multifactorial condition associated with an increased risk of CVDs. Dietary habits influence a wide range of cardiometabolic risk factors, including blood lipid and lipoprotein concentrations, obesity, hypertension, glucose–insulin homeostasis, oxidative stress, inflammation, and endothelial function, among others [1,2,3].

Although both genetic and environmental factors contribute to the etiology of AO and related cardiometabolic disorders, diet is particularly relevant because it represents a modifiable factor that can be tailored to individual needs [4]. However, evidence suggests that general recommendations regarding macronutrient and micronutrient intake do not produce the same effects in all individuals, as genetic variations may influence individual responses to dietary interventions [5].

Consequently, there is growing interest in advancing precision nutrition approaches for the prevention and management of CVDs, obesity, and associated metabolic conditions by considering the interactions among diet, genetics, and health [6].

Furthermore, substantial evidence indicates that genetic variation can influence the absorption, synthesis, utilization, and transport of nutrients and their metabolites, thereby affecting metabolic homeostasis. Characterizing gene–diet interactions involved in CVD risk may therefore provide valuable information for developing more effective strategies to promote health and well-being and may contribute to advances in precision nutrition and personalized health care.

Some researchers have developed genetic risk scores (GRSs) that integrate the effects of multiple genetic variants to predict responses to interventions [7]. However, methodological heterogeneity and differences in the number and selection of genetic variants included in these models complicate the interpretation of findings, their replication across populations, and their potential application to the prevention and management of CVDs. Addressing these limitations is particularly important for improving strategies to prevent and manage highly prevalent, complex, multifactorial conditions, including obesity, CVDs, cancer, type 2 diabetes, and neurodegenerative disorders.

Therefore, this study aimed to evaluate the effects of a nutrigenetic diet incorporating 21 genetic variants related to lipid metabolism and cardiovascular risk on anthropometric and biochemical parameters and antioxidant capacity in adults with abdominal obesity, compared with a standard diet.

## 2. Materials and Methods

### 2.1. Study Design

A parallel-group randomized controlled clinical trial was conducted over an eight-week period at the Instituto de Nutrigenética y Nutrigenómica Traslacional between March and September 2025. The study procedures were approved by the Ethics Committee of the Centro Universitario de Ciencias de la Salud, Universidad de Guadalajara (CI-05223). Furthermore, the study was registered at ClinicalTrials.gov (NCT06847243) and conducted in accordance with the 2024 Declaration of Helsinki. All subjects provided written informed consent before participating in the study. No important changes to the trial design, eligibility criteria, interventions, outcomes, or statistical analyses were made after trial commencement.

### 2.2. Study Participants

Participants were recruited through flyers and invitations posted on official social media accounts to participate in the INNUPREC study (Nutrigenetic Intervention for the Prevention of Cardiovascular Disease; from the Spanish Intervención Nutrigenética para la Prevención de Enfermedades Cardiovasculares). A virtual screening process was conducted using Google Forms to assess eligibility.

The inclusion criteria were age 30–55 years and AO, defined as a waist circumference (WC) ≥ 80 cm for women and ≥94 cm for men [8]. Participants were excluded if they had a previous diagnosis of a chronic disease, including cardiovascular or endocrine diseases, type 2 diabetes, cancer, or autoimmune disorders. Pregnant or breastfeeding women, as well as participants who were following a prescribed dietary regimen at the time of enrollment or had followed one within the three months before the start of the study, were excluded. Additionally, individuals taking lipid-lowering or hypoglycemic medications or who had consumed nutritional supplements within the three months before the study were not eligible.

### 2.3. Selection of Genetic Variants

To select the genetic variants, an initial review was conducted to identify variants associated with cardiometabolic alterations for which nutritional recommendations had previously been reported. The first selection criterion was a risk allele frequency greater than 15% in the Mexican population. The second criterion was the availability of previously reported nutritional recommendations for each variant and evidence that these recommendations produced significant changes in biomarkers associated with CVD. The third criterion was based on the molecular mechanism of each variant and its potential impact on the development of CVD.

**Figure 1:**
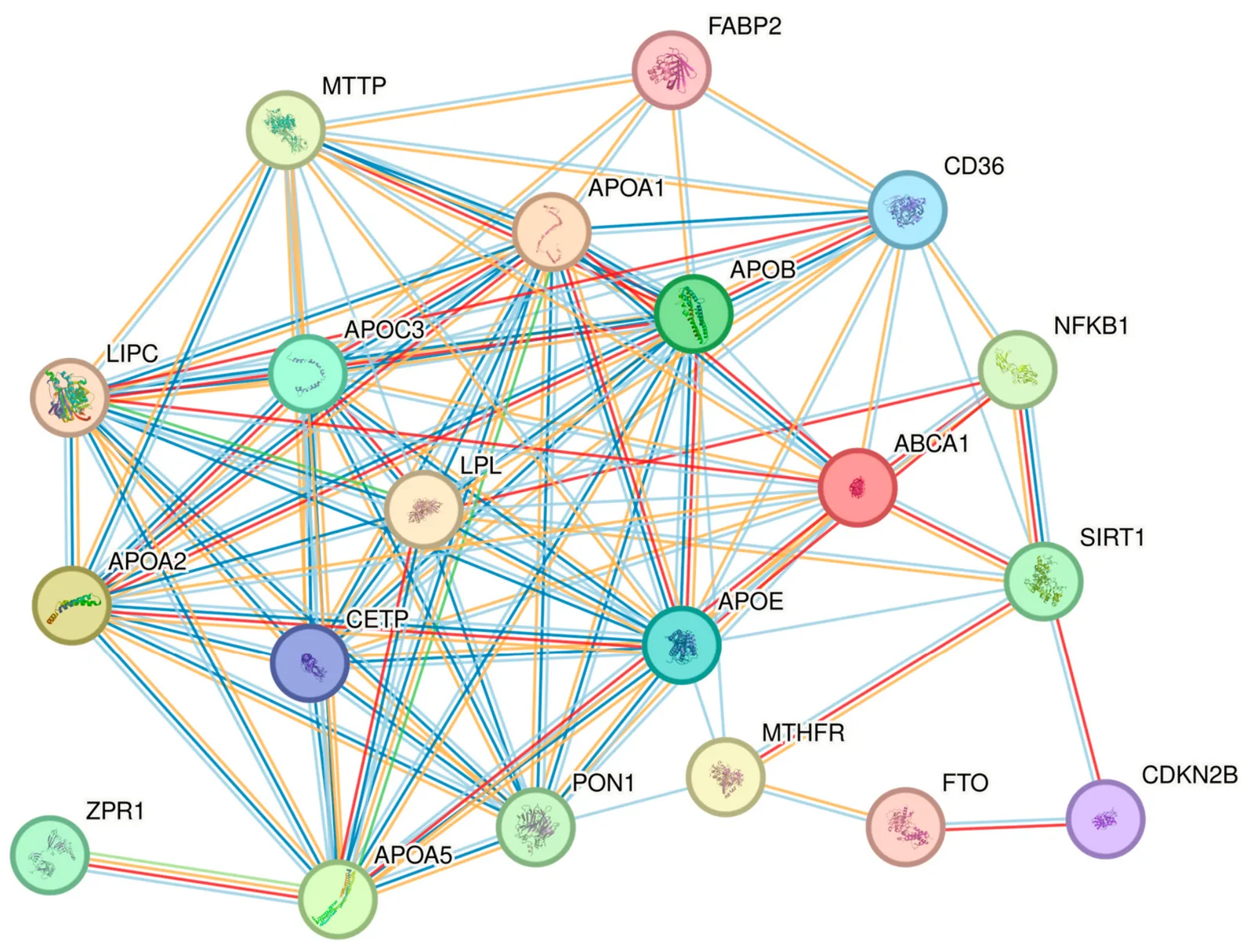
Protein–protein interaction (PPI) network of ABCA1, APOA1, APOA2, APOA5, APOB, APOC3, APOE, CD36, CETP, CDKN2B, FABP2, FTO, LIPC, LPL, MTHFR, MTTP, NFKB1, PON1, SIRT1, and ZPR1, generated via in silico analysis using the STRING database v12.0. Nodes represent individual proteins, while connecting lines (edges) represent physical and functional interactions. Nodes represent individual proteins, and connecting lines (edges) indicate physical and functional interactions. Edge colors denote the supporting evidence type: curated database entries (cyan), predicted gene fusions (red), co-occurrence (dark blue), textmining (yellow/orange) and predicted gene neighborhood (green).

Following the selection of 21 variants in 20 genes, an in silico analysis of the proteins encoded by the selected genes was performed using the STRING database v12.0 (string-db.org) to generate a predictive protein–protein interaction model based on their biological and molecular functions (Figure 1). Significant protein–protein interaction (PPI) enrichment was observed (*p* enrichment < 1.0 × 10^−16^), indicating that these proteins have more interactions with one another than would be expected for a random set of proteins of similar size and degree distribution. These findings support the biological relationship among selected genes and indicate that their encoded proteins are interconnected and can be grouped for the development of the genetic algorithm.

### 2.4. DNA Extraction and Genotyping

A peripheral blood sample was collected in EDTA tubes for the extraction of genomic DNA from leukocytes using the Roche High Pure PCR Template Preparation Kit (Roche Diagnostics, Mannheim, Germany). DNA concentration was quantified by spectrophotometry using a Multiskan SkyHigh Photometer (Thermo Scientific, Waltman, MA, USA).

The genetic variants of interest: *ABCA1* rs2230806:G>A, *APOA1* rs670:G>A, *APOA2* rs5082:T>C, *APOA5* rs662799:T>C, *APOB* rs693:C>T, *APOC3* rs5128:C>G, *APOE* rs7412:C>T and rs429358:T>C, *CETP* rs708272:G>A, *CDKN2B-AS1* rs7865618:A>G, *CD36* rs1761667:G>A, *FABP2* rs1799883:G>A, *FTO* rs9939609:T>A, *LIPC* rs1800588:C>T, *LPL* rs320:T>G, *MTTP* rs1800591:G>T, *MTFHR* rs1801133:G>T, *NFKB1* rs28362491:Ins>Del, *PON1* rs854560:A>T, *SIRT1* rs2273773:A>G, and *ZPR1* rs964184:C>G were determined using TaqMan^®^ allelic discrimination assays. Real-time PCR reactions were performed using a LightCycler^®^ 96 (Roche Diagnostics, Mannheim, Germany) under the following conditions: 95 °C for 10 min, followed by 40 cycles of denaturation at 95 °C for 15 s and annealing/extension at 60 °C for 1 min. The assays were verified using DNA samples corresponding to the three possible genotypes as positive controls, as well as negative controls. Genotyping was performed exclusively in participants assigned to the nutrigenetic diet group to guide algorithm-based dietary personalization, whereas control participants in the control group followed a standard diet without undergoing genetic testing.

### 2.5. Randomization and Allocation Concealment

Participants were randomly assigned to either the standard diet or nutrigenetic diet group using a block randomization sequence generated in R v 4.4.2 (RStudio, R Foundation for Statistical Computing, Vienna, Austria) stratified by sex and age to ensure balance between groups. To ensure allocation concealment, the randomization sequence was generated and maintained by an independent researcher who had no role in participant enrollment, clinical assessment, or dietary intervention delivery. For participants assigned to the nutrigenetic group, the specific dietary pattern (Pattern 1 or Pattern 2) was subsequently assigned according to the genetic algorithm developed as part of this study.

### 2.6. Blinding

Participants were blinded to their specific dietary group allocation (standard diet or nutrigenetic diet) and were informed only that they would receive one of two structured dietary interventions. Furthermore, laboratory personnel conducting the biochemical assays and data analysts performing the statistical evaluations were blinded to group assignments throughout the study.

### 2.7. Algorithm Design and Dietary Pattern Assignment

The genetic algorithm was applied to all participants randomly assigned to the nutrigenetic diet group to determine individual affinity percentages for two distinct nutrigenetic dietary patterns (Pattern 1 and Pattern 2). The algorithm integrated 21 genetic variants in 20 genes previously associated with lipid metabolism and CVD risk, selected based on a risk allele frequency > 15% in the Mexican population and established gene–diet interactions supported by the scientific literature. The variants were categorized into Pattern 1 or Pattern 2 according to their corresponding evidence-based nutritional recommendations (Table S1). For each participant, a composite affinity score for each dietary pattern was calculated based on three components:

1. Association Strength: The odds ratio (OR) reported in the published literature for each genetic variant in relation to CVD or metabolic risk.
2. Variant impact: A numerical value assigned to each variant proportional to its reported OR.
3. Allelic Load: The number of risk alleles present in the participant’s genotype (0 for non-risk homozygotes, 1 for heterozygotes, or 2 for risk homozygotes).

Individual affinity scores for Pattern 1 and Pattern 2 were converted into relative percentage affinities. Each participant was subsequently assigned to the dietary pattern for which they demonstrated a higher percentage affinity. No instances of equal affinity percentages were observed; in 100% of cases, a higher affinity for one specific pattern was established.

### 2.8. Intervention and Monitoring

Before randomization, all participants attended an initial visit during which their medical history was obtained, and a blood sample was collected for DNA extraction and genetic profiling. Participants were then randomly assigned to the dietary intervention groups. For participants assigned to the nutrigenetic group, the algorithm was subsequently used to determine the dietary pattern (1 or 2) with the greatest affinity to their nutrigenetic profile.

**Figure 2:**
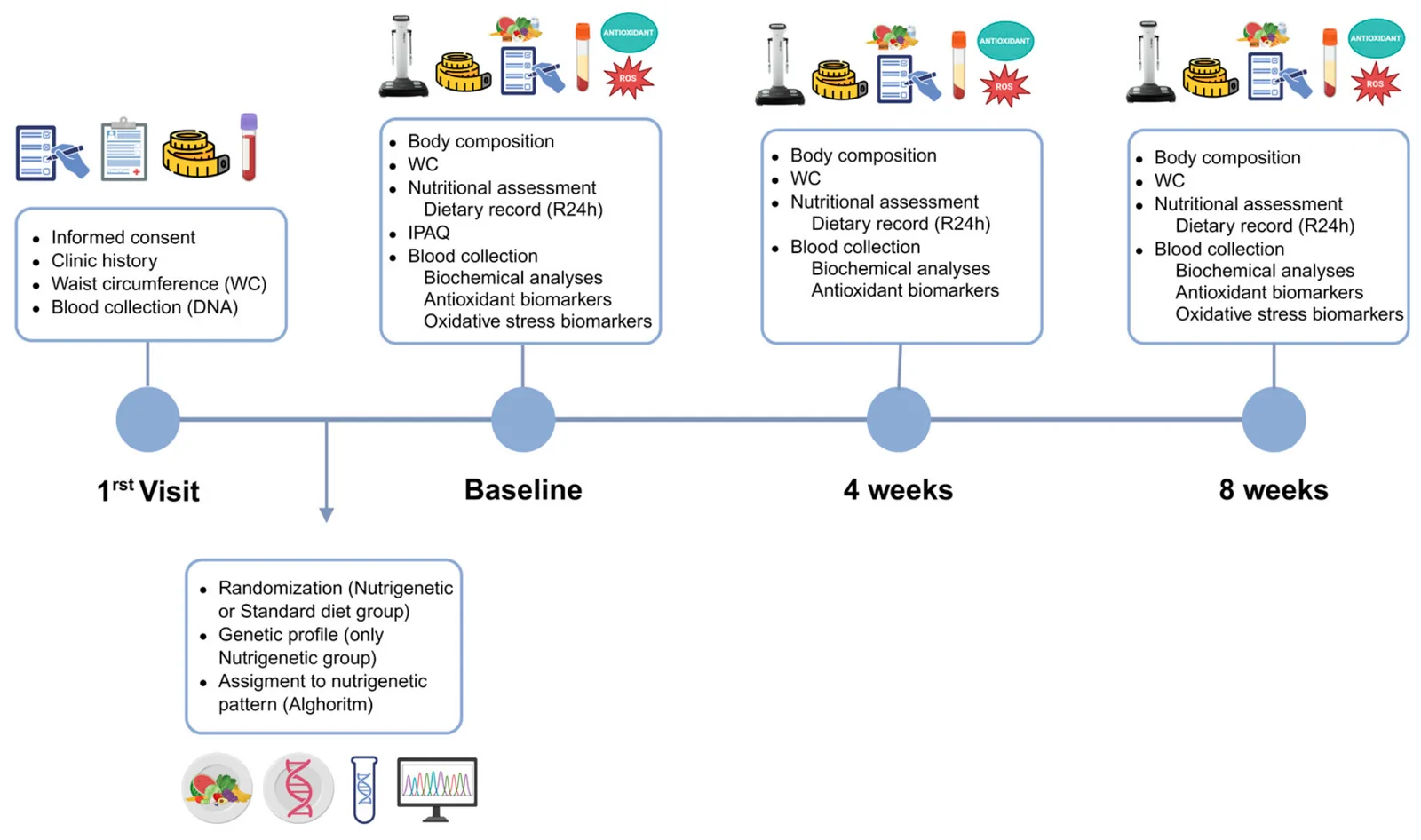
Intervention scheme. The diagram shows participant allocation, the intervention timeline, assessment points, and follow-up procedures.

The intervention consisted of an eight-week program with visits at baseline and at weeks 4 and 8. Participants were instructed to follow the dietary regimen provided through a menu designed by the research group. Both interventions were delivered by registered dietitians who received standardized training prior to study initiation to ensure protocol fidelity. Participants in both groups received personalized, structured meal plans designed by the research team according to their assigned dietary pattern. Physical activity levels were estimated at baseline using the International Physical Activity Questionnaire and assessed for changes during follow-up visits at weeks 4 and 8. To ensure intervention fidelity, dietitians conducted bi-weekly follow-up calls and face-to-face visits at baseline, week 4, and week 8 to deliver updated menus and assess compliance. All participants underwent body composition assessments and blood tests, including measurements of glucose, lipid profile, insulin, hs-CRP, and serum oxidative and antioxidant biomarkers, on three occasions: at baseline, week 4, and week 8 (final assessment) (Figure 2). Nutrient and energy intakes were determined by averaging the 24 h dietary recall records (R24h). Objective dietary adherence was defined as the percentage of adequacy (%AD), computed as the ratio of consumed daily intake (g) to recommended daily intake (g), multiplied by 100. To capture participant-perceived compliance, self-reported adherence was additionally evaluated on a 10-point ordinal rating scale, where participants rated their degree of adherence to the assigned meal plan. Adverse events (harms) were assessed non-systematically throughout the 8-week intervention. Participants were instructed to spontaneously report any unexpected symptoms, side effects, or health-related events at scheduled follow-up visits (weeks 4 and 8) or by contacting the research team between visits. Any reported adverse event was recorded and evaluated for severity (mild, moderate, or severe) and its potential relationship to the dietary intervention.

#### Nutrigenetic and Standard Diet Design

To establish the macronutrient and micronutrient composition of the nutrigenetic diets, a literature review was conducted to identify nutritional recommendations associated with each genetic variant included in the study. Based on this information, two nutrigenetic dietary patterns were defined.

The macronutrient and micronutrient distribution of the standard diet was based on the characteristics of a healthy dietary pattern according to the recommendations of the American Heart Association [9]. In addition, both nutrigenetic dietary patterns were designed to meet the recommended daily intake for the Mexican population for selected micronutrients, including vitamin C, D, and E, as well as polyphenols (Table S1) [10,11].

Specifically, Nutrigenetic Pattern 1 grouped by ten genetic variants across nine genes: *APOB* (rs693), *CDKN2B-AS1* (rs7865618), *APOA5* (rs662799), *ABCA1* (rs2230806 *SIRT1* (rs7896005), *APOA2* (rs5082), *ZPR1* (rs964184), *APOE* (rs7412 and rs429358), and *LPL* (rs320) [12,13,14,15,16,17,18,19]. In contrast, Nutrigenetic Pattern 2 consisted of eleven genetic variants across eleven genes: *CD36* (rs1761667), *FABP2* (rs1799883), *CETP* (rs708272), *FTO* (rs9939609), *APOC3* (rs5128), *MTTP* (rs1800591), *NFKB1* (rs28362491), *MTFHR* (rs1801133), *PON1* (rs854560), *LIPC* (rs1800588), and *APOA1* (rs670) [12,13,20,21,22,23,24,25,26,27,28,29].

Although fiber was not modulated by the genetic algorithm due to the lack of specific gene–fiber interaction criteria within our 21-variant panel, all intervention meal plans (the standard diet and Nutrigenetic Patterns 1 and 2) were formulated to meet the recommended daily fiber threshold (25–35 g/day). Controlling fiber content across all prescribed diets helped minimize the possibility that observed metabolic and antioxidant changes were attributable to differences in prescribed fiber intake rather than genotype-guided dietary personalization.

Energy requirements for both groups were estimated using the Mifflin-St. Jeor equation [30]. A caloric restriction of 500 kcal/day was applied to both the standard and nutrigenetic diets.

### 2.9. Anthropometric Assessment

Anthropometric measurements were obtained after a 10–12 h fast, with participants barefoot and wearing light clothing. Height was measured using a stadiometer with a precision of 1 mm (Rochester Clinical Research, Rochester, NY, USA), with the participant’s head in the Frankfurt plane, arms alongside the body, and shoulders, buttocks, and heels touching the device. Waist circumference was measured using a Lufkin Executive^®^ tape with a range of 0–200 cm and a precision of 0.1 cm (New Brighton, MN, USA).

Tetrapolar bioelectrical impedance analysis was used to assess body composition using an InBody 370 analyzer (Biospace Co., Seoul, Republic of Korea). Anthropometric parameters were evaluated three times during the intervention: at baseline and at weeks 4 and 8 (Figure 2).

### 2.10. Dietary Intake Analysis

Dietary intake was assessed using a food record representing a habitual day, including the quantity and description of foods consumed; the records were analyzed using Nutritionist Pro™ Diet Analysis software v7.9 (Axxya Systems, Stafford, TX, USA). To improve the accuracy of dietary intake estimates, food scales and models from Nasco^®^ (Fort Atkinson, WI, USA), as well as household measures such as cups and spoons, were used.

Dietary *n*-3 intake was estimated as the sum of the following polyunsaturated fatty acids (PUFAs): alpha-linolenic acid (18:3), stearidonic acid (18:4), eicosapentaenoic acid (20:5), docosapentaenoic acid (22:5), and docosahexaenoic acid (22:6). Dietary *n*-6 intake was calculated as the sum of linoleic acid (18:2), and arachidonic acid (20:4). The *n*-6:*n*-3 PUFA ratio was determined by dividing total *n*-6 intake by total *n*-3 intake (g/day). A ratio ≤5:1 was considered adequate [31].

Micronutrient intake was compared with the reference values established for the Mexican population by the National Institute of Medical Sciences and Nutrition “Salvador Zubirán” and with NOM-051-SCFI/SSA1-2010 [10,11].

### 2.11. Biochemical Measurements

Peripheral blood samples were collected by venipuncture in the morning after a 10–12 h fast using a BD Vacutainer^®^ system (Becton, Dickinson and Company, NJ, USA). Serum was separated by centrifugation at 4 °C for 15 min at 3500 rpm and stored at −80 °C until analysis.

Serum glucose, triglycerides (TG), total cholesterol (TC), and high-density lipoprotein cholesterol (HDL-c) were measured by dry chemistry using a Vitros 350 Analyzer (Ortho-Clinical Diagnostics, Johnson & Johnson Services Inc., Rochester, NY, USA). Low-density lipoprotein cholesterol (LDL-c) was calculated using the Friedewald formula when TG concentrations were <400 mg/dL. Very-low-density lipoprotein cholesterol (VLDL-c) was estimated by dividing total TG by 5. Insulin concentrations were determined using a chemiluminescent immunoassay (Diasorin, Liaison^®^, Milan, Italy).

High-sensitivity *C*-reactive protein (hs-CRP) concentrations were determined using the BA 400 *C*-reactive protein high-sensitivity assay kit (No. 22927; BioSystems S.A., Barcelona, Spain) on a Mindray BS-120 clinical chemistry analyzer (Mindray, Shenzhen, China), according to the manufacturer’s instructions. The assay is based on a latex-enhanced turbidimetric method in which latex particles coated with specific anti-human CRP antibodies agglutinate in the presence of CRP in the sample, producing a change in absorbance proportional to the CRP concentration.

### 2.12. Definition of Cardiometabolic Disorders

Cardiometabolic disorders were defined according to the criteria established by the National Cholesterol Education Program Adult Treatment Panel III (NCEP-ATP III) for hypercholesterolemia, hypertriglyceridemia, hypoalphalipoproteinemia, and elevated LDL-c [32].

The triglyceride-to-HDL cholesterol (TG/HDL-c) ratio was evaluated using sex-specific thresholds (150/40 for men and 150/50 for women), corresponding to the limits above which TG and HDL-c concentrations are considered inadequate. Castelli Risk Index I (CRI-I) was calculated by dividing TC by HDL-c, whereas Castelli Risk Index II (CRI-II) was calculated by dividing LDL-c by HDL-c [33].

Insulin resistance was estimated using the Homeostasis Model Assessment of Insulin Resistance (HOMA-IR), calculated as fasting glucose (mg/dL) × fasting insulin (µIU/mL)/405. The triglyceride-glucose (TyG) index was calculated as ln[(fasting glucose (mg/dL) × triglycerides mg/dL)/2] [34]. The cutoff values used to define cardiometabolic disorders are presented in Table S2.

### 2.13. Antioxidant Biomarkers

Total antioxidant capacity (TAC) was determined using a colorimetric assay with the OxiSelect™ Total Antioxidant Capacity (TAC) Assay Kit (STA-360; Cell Biolabs, San Diego, CA, USA), according to the manufacturer’s instructions. The assay is based on a single electron transfer (SET) mechanism in which Cu^2+^ is reduced to Cu^+^ in the presence of antioxidants. The reduced copper ion subsequently reacts with a chromogenic substrate, generating a color signal proportional to the total antioxidant capacity.

Serum glutathione peroxidase (GPx) activity was determined using the Glutathione Peroxidase Assay Kit (No. 703102; Cayman Chemical Company, Ann Arbor, MI, USA). GPx activity was measured indirectly through a coupled reaction with glutathione reductase, in which NADPH is oxidized to NADP^+^.

Superoxide dismutase (SOD) activity was measured using the OxiSelect™ Superoxide Dismutase Activity Assay Kit (STA-340; Cell Biolabs, San Diego, CA, USA). This assay is based on the generation of superoxide anions by the xanthine/xanthine oxidase system, which reduces a chromogen to form a water-soluble formazan dye. SOD activity was determined based on the degree of inhibition of chromogen reduction.

Catalase activity was determined using the OxiSelect™ Catalase Activity Assay Kit (STA-341; Cell Biolabs, San Diego, CA, USA) based on a colorimetric method. Quantification was performed using a Thermo Fisher Scientific Multiskan SkyHigh Microplate Photometer (Waltham, MA, USA).

### 2.14. Oxidative Damage Biomarkers

Serum lipid peroxidation (LPO) levels were measured using the Lipid Peroxidation Assay Kit (Oxford Biomedical Research Inc., Rochester Hills, MI, USA), which is based on the reaction between the chromogenic reagent *N*-methyl-2-phenylindole (R1) and malondialdehyde (MDA).

Serum 8-isoprostane levels were measured using the Human 8-Isoprostane enzyme-linked immunosorbent assay (ELISA) kit (Cat. No. MBS3802509; MyBioSource Inc., San Diego, CA, USA), according to the manufacturer’s instructions. Serum levels of oxidized high-density lipoprotein (oxHDL) and oxidized low-density lipoprotein (oxLDL) were measured using ELISA kits according to the manufacturer’s protocols (Cell Biolabs, Catalog No. STA-888 and STA-369, respectively; San Diego, CA, USA). Quantification was performed using a Thermo Fisher Scientific Multiskan SkyHigh Microplate Photometer (Waltham, MA, USA).

### 2.15. Statistical Analysis

The sample size was calculated a priori using OpenEpi software (v.3.01) based on the post-intervention insulin differences reported by de Luis et al. [28]. Assuming a two-sided significance level (α) of 5% and a power (1-β) of 80%, a minimum of 25 participants per group was required. To account for an anticipated 20% dropout rate, the sample size was increased to 30 participants per group. Data normality was assessed using the Shapiro–Wilk test. Normally distributed continuous variables were expressed as mean ± standard deviation (SD), whereas non-normally distributed variables were reported as median and interquartile range (IQR). Categorical variables were presented as frequencies and percentages.

For comparisons between two independent groups, Student’s *t*-test was used for normally distributed variables and the Mann–Whitney U test for non-normally distributed variables. For related samples, the paired Student’s *t*-test and Wilcoxon signed-rank test were used for normally and non-normally distributed variables, respectively. Differences in categorical variables between groups were evaluated using the chi-square test, whereas within-group changes from baseline to post-intervention were assessed using McNemar’s test.

Univariate general linear models (ANCOVA), adjusted for baseline measurements, age, and sex, were used to evaluate treatment effects on quantitative variables. To limit type I error inflation due to multiplicity across endpoints, raw *p*-values derived from these models were corrected using the Benjamini–Hochberg false discovery rate (FDR) procedure. Point estimates of treatment effects are reported as baseline-adjusted between-group mean differences with 95% CIs, accompanied by both raw *p*-values and FDR-adjusted *p*-values (q-values). All statistical analyses were performed using IBM SPSS Statistics for Windows, version 28.0 (IBM Corp., Armonk, NY, USA). A *p*-value < 0.05 was considered statistically significant.

All prespecified baseline and follow-up determinations were conducted according to the original plan, and the trial concluded successfully as scheduled without early termination due to adverse events.

Primary outcome (body fat mass) and secondary outcomes (body weight, body fat percentage, WC, insulin, HOMA-IR, hs-CRP, catalase, SOD, and GPx) were analyzed on a per-protocol (PP) basis, including only participants who completed the 8-week intervention and attended all follow-up visits. Missing data due to dropouts or missed visits were not imputed, and a complete-case analysis was conducted for all time points (baseline, week 4, and week 8).

## 3. Results

### 3.1. Clinical, Anthropometric and Biochemical Characteristics of the Study Participants

**Figure 3:**
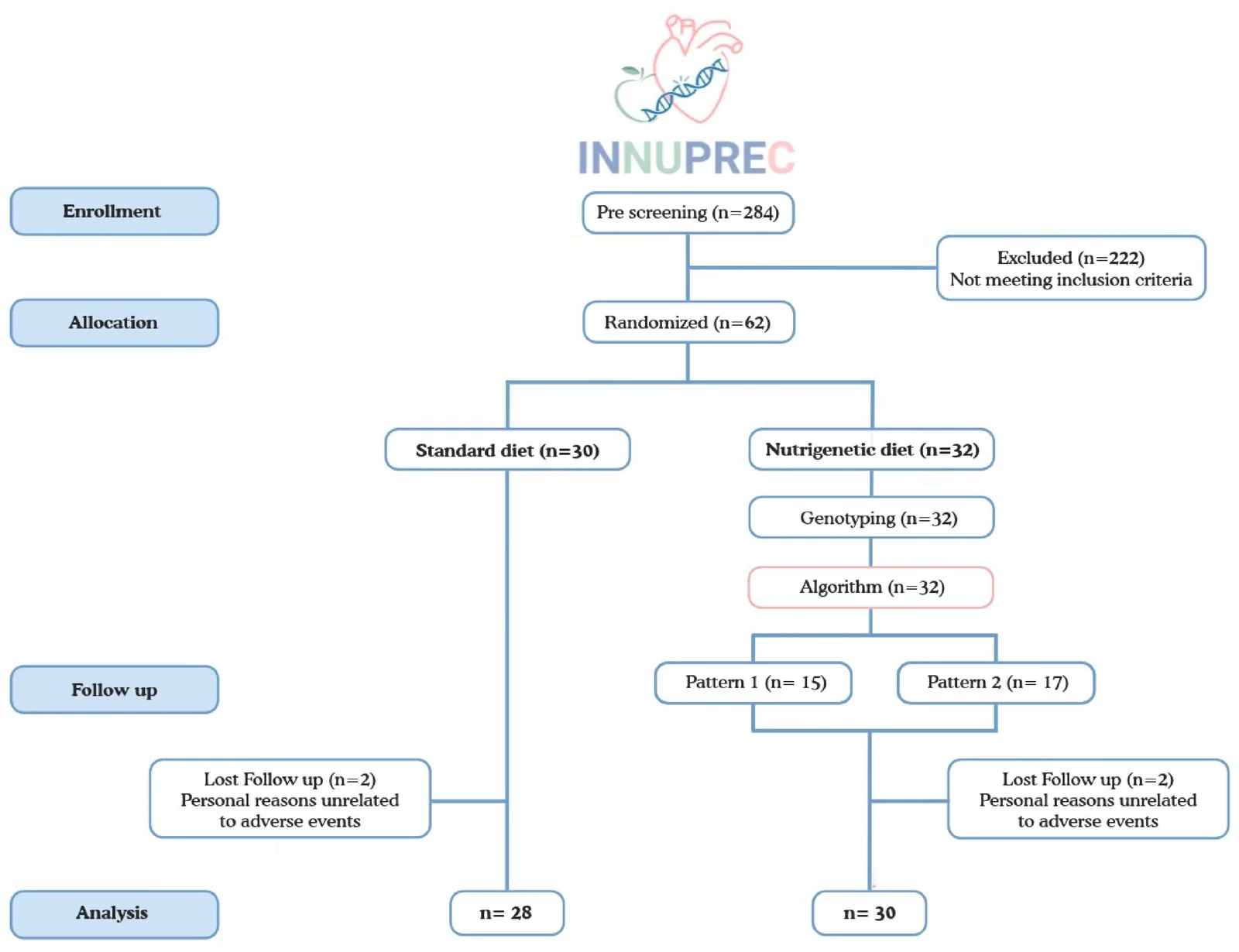
Flow diagram.

A total of 62 participants were enrolled and randomly assigned to either the standard diet group (*n* = 30) or the nutrigenetic diet group (*n* = 32). Within the nutrigenetic group, participants were assigned to personalized dietary recommendations based on their genetic profile, with 15 participants assigned to Pattern 1 and 17 to Pattern 2. A total of 4 participants (*n* = 2 in the standard group and *n* = 2 in the nutrigenetic group) discontinued the trial prior to the week-8 follow-up for personal reasons (time constraints and personal scheduling conflicts). None of the dropouts were related to dietary dissatisfaction, study logistics, or adverse events. A total of 58 participants completed the 8-week trial and were included in the final per-protocol analysis in the INNUPREC study (Figure 3).

The mean age of the participants was 41.0 ± 8.2 years (41.5 ± 8.0 years in the standard diet group vs. 40.5 ± 8.5 years in the nutrigenetic diet group, *p* = 0.670). Overall, 72.4% of the participants were women (*n* = 42), and 27.6% were men (*n* = 16). No significant differences between groups were observed in baseline clinical characteristics (Table 1). Genotypic and allelic frequencies for all evaluated genetic variants in the nutrigenetic intervention group were assessed and are fully reported in Supplementary Table S3.

Baseline anthropometric and biochemical variables are shown in Table 2. Regarding anthropometric characteristics, mean body fat percentage and BMI were above the recommended values. Regarding the biochemical profile, mean glucose, CT, TG, LDL-c, VLDL-c, CRI-I (TC/HDL-c), TG/HDL-c ratio, CRI-II (LDL-c/HDL-c), TyG Index, and HOMA-IR values were above the recommended levels, while mean HDL-c levels were below the recommended level in women. No significant differences between groups were observed at baseline, indicating similar anthropometric and biochemical characteristics before the intervention.

**Figure 4:**
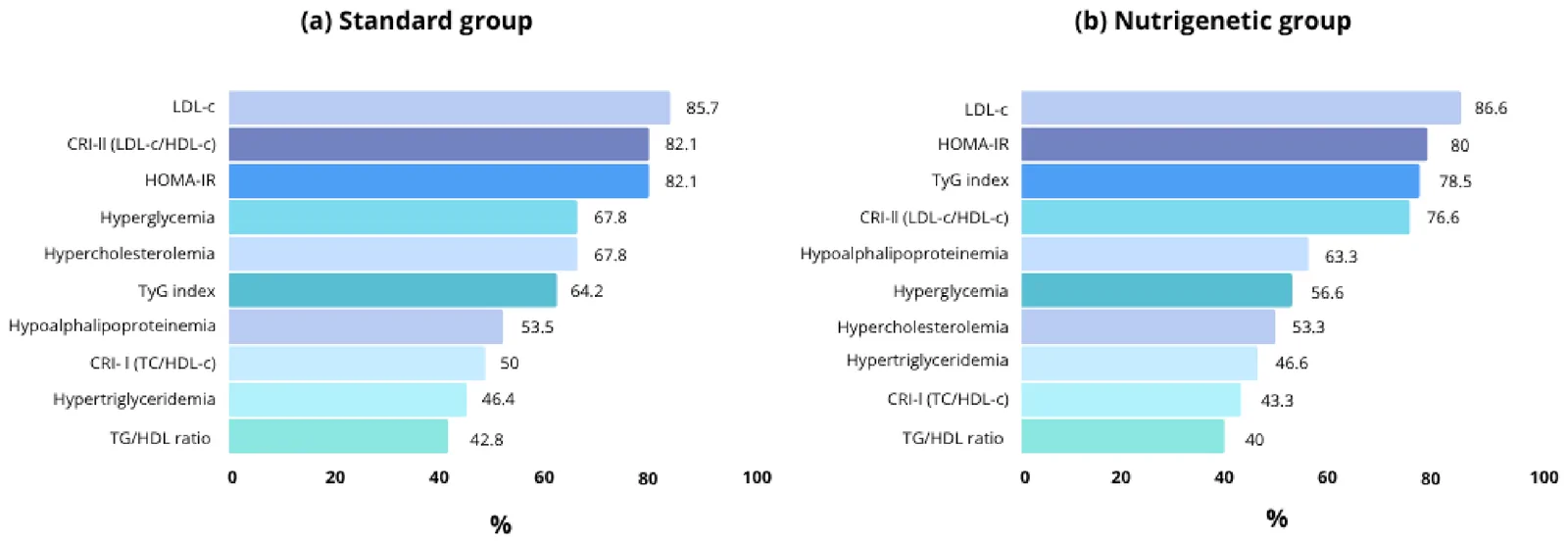
Prevalence of cardiometabolic disorders in the study groups at baseline. (**a**) Prevalence of cardiometabolic disorders in the standard diet group; (**b**) prevalence of cardiometabolic disorders in the nutrigenetic diet group. LDL-c: low-density lipoprotein cholesterol; TG: triglycerides; HDL-c: high-density lipoprotein cholesterol; TC: total cholesterol; CRI-I: Castelli Risk Index I; CRI-II: Castelli Risk Index II; TyG index: triglyceride-glucose index; HOMA IR: Homeostasis Model Assessment of Insulin Resistance.

The most prevalent cardiometabolic disorders in both groups were elevated LDL-c and HOMA-IR, both of which are associated with increased cardiovascular risk (Figure 4). Overall, a high prevalence of cardiometabolic abnormalities was observed; however, no significant differences were found between groups at baseline.

### 3.2. Baseline Dietary Characteristics of the Study Groups

Dietary intake in both groups before the intervention is shown in Table 3. Regarding dietary characteristics, a high prevalence of excessive intake of total fatty acids (TFA), saturated fatty acids (SFA), and cholesterol was observed, as well as a high *n*-6:*n*-3 polyunsaturated fatty acid (PUFA) ratio. In addition, PUFA and fiber intake were below the recommended levels. However, the baseline nutritional analysis showed no statistically significant differences between groups.

### 3.3. Changes in Anthropometric and Biochemical Variables in the Study Groups

Changes in anthropometric variables throughout the intervention are shown in Table 4. Analysis of dietary intake during follow-up revealed slightly higher protein consumption in the standard diet group, whereas the nutrigenetic group exhibited a marginally higher intake of carbohydrates and saturated fats (Supplementary Materials, Table S4). Despite these minor macronutrient variations, self-reported dietary adherence was equivalent and consistently high (~80%) across both intervention arms (Supplementary Materials, Table S5).

At the end of the intervention, both groups showed statistically significant reductions in body weight and waist circumference. Body fat mass also decreased significantly in both groups. In addition, the nutrigenetic diet group showed significant reductions in body fat percentage and BMI. Comparison of the changes (Δ) between groups showed a significantly greater reduction in body weight in the nutrigenetic diet group than in the standard diet group (*p* = 0.045). A greater reduction in body fat mass was also observed in the nutrigenetic diet group, although the between-group difference did not reach statistical significance (*p* = 0.085). When fat mass changes were analyzed by sex, women in the nutrigenetic diet group showed a greater reduction than women in the standard diet group, although this difference was also not statistically significant (*p* = 0.056).

Biochemical changes throughout the intervention are shown in Table 5. At the end of the intervention, participation in the nutrigenetic diet group showed statistically significant reductions in insulin, HOMA-IR, and hs-CRP compared with baseline. No statistically significant differences in the changes in these variables were observed between the two dietary groups.

Changes in the prevalence of cardiometabolic disorders after the nutritional intervention are shown in Table 6. In the nutrigenetic diet group, significant reductions were observed in the prevalence of elevated LDL-c, an elevated TyG index, and HOMA-IR. However, the differences in prevalence changes between the standard and nutrigenetic diet groups were not statistically significant.

Changes in antioxidant and oxidative damage biomarkers are shown in Table 7. At the end of the nutritional intervention, the nutrigenetic diet group showed significantly greater increases in catalase and GPx activity than the standard diet group. Within the nutrigenetic diet group, significant increases in catalase, GPx, and SOD activity and total antioxidant capacity were observed, together with significant reductions in LPO and 8-isoprostane levels compared with baseline. The standard diet group also showed significant increases in catalase and SOD activity and total antioxidant capacity, as well as a significant reduction in 8-isoprostane levels compared with baseline.

### 3.4. Baseline-Adjusted Treatment Effects and Correction for Multiple Comparisons

To address potential baseline imbalances and account for multiple statistical tests across prespecified endpoints, univariate general linear models (ANCOVA) adjusted for baseline values, age, and sex were used for the primary outcome (BF) and secondary outcomes (Table 8). Treatment effects are presented as baseline-adjusted between-group differences with 95% confidence intervals (95% CIs). To control the false discovery rate (FDR) associated with multiple testing, *p*-values were adjusted using the Benjamini–Hochberg (BH) procedure.

In the baseline-adjusted ANCOVA models prior to FDR adjustment, statistically significant between-group differences were observed for fasting insulin, HOMA-IR, hs-CRP, and catalase activity. Following Benjamini–Hochberg FDR correction, the differences between groups remained statistically significant for insulin, HOMA-IR, and hs-CRP. Catalase activity showed an initial increase in the nutrigenetic group, but this difference did not remain statistically significant after FDR correction. Furthermore, no statistically significant differences were observed between groups for the primary outcome, body fat mass (kg), or secondary outcomes, including body weight, body fat percentage, and GPx activity.

### 3.5. Safety and Adverse Events

Both dietary interventions (standard diet and nutrigenetic diet) were well tolerated by all study participants throughout the 8-week intervention period. No mild, moderate, or severe adverse events, side effects, or safety-related issues were reported during the trial.

## 4. Discussion

In recent years, AO has been consistently recognized as a major risk factor for the development of cardiometabolic disorders. The increased risk of CVDs in individuals with AO is closely associated with a cluster of metabolic alterations, including dyslipidemia, hyperglycemia, insulin resistance, elevated oxidative stress, and a concomitant reduction in antioxidant capacity. These disturbances are further influenced by a combination of modifiable lifestyle factors, such as an atherogenic diet, physical inactivity, alcohol consumption, and smoking, as well as genetic predisposition [35]. Notably, accumulating evidence indicates that standard dietary recommendations do not produce uniform metabolic responses across individuals, as dietary exposures interact dynamically with each person’s genetic background [36]. This biological heterogeneity underscores the need for precision nutrition strategies that account for individual genetic variability when designing dietary interventions. Therefore, the aim of this study was to evaluate the effect of a nutrigenetic diet tailored according to 21 genetic variants associated with lipid metabolism and cardiovascular risk on anthropometric parameters, biochemical markers, and antioxidant capacity in participants with AO, compared with a standard dietary intervention.

At baseline, participants in both groups exhibited a characteristically atherogenic dietary pattern. This profile was defined by a high intake of TFA, SFA, and cholesterol, along with an elevated *n*-6:*n*-3 PUFA ratio and insufficient total intake of PUFAs and dietary fiber. Such a dietary profile has been consistently associated with increased oxidative damage and a chronic low-grade inflammatory state, both of which accelerate the progression of AO and its related comorbidities [37]. These findings are similar to those reported by Campos-Pérez et al. in a Mexican population, in which individuals with excess body weight showed high intakes of TFA (36.9 ± 7.1%) and SFA (12.2 ± 3.5%), along with inadequate PUFA intake (6.7 ± 3%). Regarding the *n*-6:*n*-3 PUFA ratio, we observed a lower ratio than previously reported (8.1:1 vs. 12:1); however, it remained substantially above the current nutritional recommendation of 5:1, indicating an imbalance in fatty acid intake [38]. Similarly, dietary fiber intake among our participants was below the recommended levels (25–35 g/day), a deficit that may contribute to impaired glucose and lipid homeostasis, as well as unfavorable changes in gut microbiota composition [39].

Consistent with this adverse dietary context, a high baseline prevalence of cardiometabolic alterations was observed. More than half of the study participants had elevated LDL-c, an increased CRI-II (LDL-c/HDL-c), insulin resistance assessed by both HOMA-IR and the TyG index, hyperglycemia, hypercholesterolemia, and hypoalphalipoproteinemia. These prevalences were higher than those reported for the Mexican national population in ENSANUT 2022, which reported prevalences of hypercholesterolemia, hypertriglyceridemia, and hypoalphalipoproteinemia of 30.6%, 57.3%, and 26.3%, respectively [2]. This discrepancy may reflect the metabolically vulnerable phenotype of individuals with AO, which is commonly associated with clusters of alterations that increase cardiovascular risk. Consequently, these data highlight the potential clinical value of precision nutrition strategies tailored to this population.

Following the nutritional intervention, one of the most relevant findings was that participants following the nutrigenetic diet achieved a significantly greater reduction in body weight than those assigned to the standard diet. Greater reductions in total fat mass and body fat percentage were also observed in the nutrigenetic group, although the between-group differences did not reach statistical significance. Notably, women in the nutrigenetic diet group showed greater reductions in fat mass and body fat percentage than women in the standard diet group, although these differences were not statistically significant. This finding is of interest given that women may exhibit attenuated responses to weight-loss interventions due to sex-related differences in energy metabolism and endocTrine regulation [40,41].

From a physiological perspective, women generally have a lower proportion of lean body mass, which translates into a lower basal metabolic rate and, consequently, lower substrate oxidation and energy expenditure during caloric restriction. Moreover, in response to an energy deficit, the female biological system may tend to preserve energy stores through adaptive thermogenesis, involving a downregulation of resting energy expenditure aimed at maintaining energy balance. Several controlled studies have demonstrated that such sexual dimorphism in energy metabolism may result in attenuated weight-loss responses in women compared with men under comparable dietary restrictions [42,43,44]. Within this context, the trend toward a greater reduction in fat mass observed among women in the nutrigenetic group suggests that incorporating genetic information into dietary planning may help optimize individual responses to weight-loss strategies. However, any exploratory observations by sex must be strictly contextualized as descriptive and hypothesis-generating due to the low statistical power within the male subgroup.

In addition, both groups exhibited a significant within-group reduction in WC by the end of the intervention, indicating a general improvement in central adiposity associated with caloric restriction. However, only the nutrigenetic diet group achieved a significant within-group decrease in BMI. These findings suggest a potential additional benefit of precision nutrition strategies over generalized approaches. These results strengthen the consensus that precision nutrigenetic models can potentiate the metabolic benefits of dietary interventions, particularly within subpopulations, such as women, who face steep physiological barriers to effective fat mass reduction.

Previous clinical trials have likewise demonstrated that nutrigenetic interventions may enhance weight-loss outcomes and optimize metabolic phenotypes compared with conventional hypocaloric diets. Randomized controlled trials have reported that dietary regimens personalized according to genetic variants involved in energy metabolism, lipid transport, and appetite regulation, such as *FTO* (rs9939609), *APOA2* (rs5082), *APOA5* (rs662799), *CETP* (rs708272), *LPL* (rs320), *FABP2* (rs1799883), and *CD36* (rs1761667), may lead to greater reductions in body weight, fat mass, and WC than conventional dietary interventions. These genotype-driven modifications in macronutrient distribution and food selection may optimize lipid oxidation, insulin sensitivity, and satiety signaling, thereby promoting metabolic efficiency and long-term dietary adherence [45,46,47].

In alignment with our findings, Pérez-Beltrán et al. conducted a nutrigenetic intervention in Mexican adults with obesity using an 11-genetic-variant panel and reported that the nutrigenetic diet group achieved better outcomes in body weight, BMI, fat mass, and body fat percentage than the control group. They concluded that nutrigenetic approaches may be associated with improved body composition responses in populations with obesity [48]. Complementary evidence from a Dutch randomized controlled trial employing a seven-variant nutrigenetic profile over nine weeks further supports these insights; participants assigned to the nutrigenetic diet achieved significantly greater reductions in body fat percentage (−1.1 [0.1 to −2.3] vs. +0.6 [0.5 to 1.7]%) and WC than those in the control group [49].

Consistent observations have emerged from broader precision nutrition research networks. In the European Food4Me study, personalized advice guided by genotype was associated with healthier dietary behavior, greater long-term adherence, and improved body composition outcomes relative to generalized dietary guidelines [45,46]. Similarly, the NOW Trial demonstrated that integrating genetic data into nutritional counseling may improve outcomes related to body weight and fat mass management compared with standard advice [47]. Collectively, these findings add to the growing evidence suggesting that genotype-guided nutrition may promote favorable anthropometric and metabolic responses. Taken together, data from diverse populations suggest that nutrigenetic approaches may have clinical value within precision nutrition, offering an individualized framework for obesity management [23,46,50,51].

Regarding biochemical parameters, participants in the nutrigenetic diet group exhibited significant within-group reductions in fasting insulin concentrations and HOMA-IR values after the intervention, whereas no significant within-group changes were observed in the standard diet group. Furthermore, in baseline-adjusted ANCOVA models controlling for baseline levels, age, and sex, and after applying the Benjamini–Hochberg false discovery rate (FDR) correction for multiple testing, between-group comparisons confirmed statistically significant differences favoring the nutrigenetic group for both fasting insulin [−3.86 (95% CI: −6.00 to −1.73), *p* = 0.005], HOMA-IR [−1.03 (95% CI: −1.65 to −0.42), FDR-adjusted *p* = 0.005]. In addition, greater reductions in the prevalence of elevated HOMA-IR (33.3% vs. 10.7%) and elevated TyG index (20% vs. 10.8%) were observed in the nutrigenetic diet group compared with the standard diet group. The TyG index has emerged as a useful surrogate marker of insulin resistance and cardiometabolic risk. The favorable baseline-adjusted changes in insulin sensitivity observed in our study may be related to the individualized macronutrient distributions informed by variants in *FTO*, *LPL*, *LIPC*, *SIRT1*, *APOA2*, *FABP2*, and *MTTP*, genes involved in glucose–insulin homeostasis, lipid metabolism, and adipose tissue function [21,22,26,52,53,54,55].

To our knowledge, few nutrigenetic interventions have specifically reported reductions in fasting insulin concentrations as an outcome. A relevant precedent is the study by Pérez-Beltrán et al., in which both the control and nutrigenetic diets were associated with comparable reductions in insulin concentrations (Δ control diet: −4.55 ± 1.7, Δ nutrigenetic diet: −4.66 ± 1.4), suggesting that caloric restriction may itself be sufficient to improve insulin concentrations, potentially attenuating the additional effect of nutrigenetic personalization [49]. These findings are consistent with evidence from clinical trials showing that dietary patterns such as Mediterranean and high-protein, low-glycemic-index diets, when implemented with caloric restriction, are associated with lower fasting insulin concentrations and improved insulin sensitivity in individuals with obesity [56,57].

The clinical relevance of these findings extends beyond glucose homeostasis. Sustained insulin resistance is known to activate NFκB and JNK signaling cascades within adipose tissue and the vascular endothelium, fostering a chronic pro-inflammatory environment that contributes to endothelial dysfunction and atherogenesis [58,59]. In this context, the reduction in estimated insulin resistance observed with the nutrigenetic intervention may represent a physiologically relevant benefit. This improvement could influence metabolic, lipid, and inflammatory pathways involved in cardiometabolic risk, supporting the potential value of nutrigenetic dietary interventions in this population.

Elevated hs-CRP concentrations are widely recognized as predictors of atherosclerotic and cardiovascular risk, even in individuals without overt lipid abnormalities. In the present study, participants following the nutrigenetic diet achieved a significant reduction in hs-CRP concentrations (−4.34 ± 6.8 mg/L, *p* = 0.001). Moreover, in baseline-adjusted ANCOVA models controlling for baseline levels, age, and sex and following Benjamini–Hochberg false discovery rate (FDR) adjustment for multiple testing, between-group comparisons confirmed a statistically greater reduction in hs-CRP in the nutrigenetic group (−2.29 [95% CI −3.85 to −0.73], *p* = 0.015). This finding suggests an attenuation of the chronic low-grade inflammation that characterizes AO. It is also consistent with previous precision nutrition trials suggesting that genotype-based dietary modulation may influence inflammatory biomarkers and complement the anti-inflammatory effects associated with caloric restriction [48,60,61].

Mechanistically, the decrease in hs-CRP observed in the nutrigenetic diet group may be related to the modulation of NFκB and JNK signaling pathways. These pathways are involved in the regulation of pro-inflammatory cytokines such as TNF-α and IL-6, which contribute to hepatic hs-CRP synthesis [58,59]. Concurrently, attenuation of systemic inflammation may benefit endothelial and lipoprotein function by supporting endothelial nitric oxide synthase (eNOS) activity, preserving nitric oxide bioavailability, enhancing HDL-c functionality, and protecting LDL-c from oxidative modification, thereby promoting a less atherogenic vascular environment [62,63,64]. Given that hs-CRP synthesis is largely driven by IL-6 signaling and local adipose tissue inflammation, our findings suggest that nutrigenetic approaches may contribute to lowering hs-CRP concentrations, particularly when combined with caloric restriction and subsequent weight loss. Although direct evidence from nutrigenetic trials examining hs-CRP remains limited, randomized trials using personalized dietary interventions have reported significant reductions in downstream IL-6 and TNF-α [48,60,61], providing a plausible mechanistic link to the hs-CRP reductions observed in our study. Overall, these results highlight the potential of nutrigenetic interventions to favorably influence metabolic homeostasis by targeting both insulin sensitivity and systemic inflammation.

Regarding biomarkers of antioxidant enzyme activity and oxidative damage, participants assigned to the nutrigenetic diet exhibited significant increases in catalase, GPx, SOD activity and TAC at the end of the intervention. When comparing delta values between groups, participants following the nutrigenetic diet showed significantly greater increases in both catalase and GPx activity (5.16 ± 6.4 vs. 2.65 ± 1.63 U/mL, *p* = 0.040; 0.14 ± 0.1 vs. 0.04 ± 0.2 U/mL, *p* = 0.043, respectively) than those following the standard diet. However, in baseline-adjusted ANCOVA models, catalase activity showed a greater increase in the nutrigenetic group, although the difference was no longer statistically significant after FDR correction. In contrast, GPx did not show a statistically significant between-group difference after correction for multiple testing.

At the molecular level, the greater increases in catalase and GPx activity in the nutrigenetic group suggest more efficient activation of peroxide-detoxifying pathways. These enzymes act downstream of SOD to neutralize hydrogen peroxide, preventing its accumulation and limiting hydroxyl radical formation, lipid peroxidation, and redox-sensitive inflammatory signaling. This enhanced antioxidant response may be related to the functional interaction between several genetic variants included in our panel and redox-sensitive pathways modulated by essential micronutrients. Genes such as *NFKB1*, *SIRT1*, *PON1*, and *MTHFR* are linked to pathways involved in inflammatory regulation, antioxidant gene expression, protection of HDL-c against oxidation, and homocysteine-related oxidative stress. Genotype-specific differences in micronutrient metabolism may therefore contribute to the greater enzymatic response observed in the nutrigenetic group. By tailoring dietary targets to these genetic differences, the intervention may have optimized antioxidant defenses beyond those expected from caloric restriction alone.

Complementing the improvements in antioxidant enzyme activity, the nutrigenetic group showed significant reductions in LPO and 8-isoprostane, whereas the standard diet group showed a significant reduction only in 8-isoprostane. The additional decrease in LPO observed in the nutrigenetic group suggests greater attenuation of oxidative damage to lipids and is consistent with the improvements observed in antioxidant enzyme activity. Mechanistically, LPO and 8-isoprostane represent complementary indicators of oxidative lipid damage. LPO reflects free radical-initiated chain reactions affecting PUFAs within cellular membranes, whereas 8-isoprostane is a prostaglandin F_2_α isomer generated nonenzymatically from arachidonic acid and is considered a reliable in vivo biomarker of free radical-mediated lipid peroxidation [65,66]. The significant reductions in both biomarkers within the nutrigenetic group, compared with the reduction in 8-isoprostane alone in the standard group, suggest a broader improvement in oxidative lipid damage.

Taken together, these findings indicate a biologically plausible redox response to the nutrigenetic diet. SOD catalyzes the initial dismutation of the superoxide anion (O_2_•^−^) into hydrogen peroxide (H_2_O_2_), making catalase and GPx important downstream enzymes for the resolution of oxidative stress. The greater increases in catalase and GPx observed in the nutrigenetic diet group, together with the reductions in LPO and 8-isoprostane, suggest enhanced antioxidant defense. Interestingly, although the standard diet group showed improvements in catalase, SOD, and TAC, it did not exhibit a significant reduction in LPO. This finding suggests that caloric restriction itself may improve antioxidant defenses, while the nutrigenetic intervention may provide additional benefits for specific antioxidant pathways.

Interestingly, although self-reported dietary adherence scores were slightly lower in the nutrigenetic group compared with the standard diet group, the personalized intervention yielded statistically significantly greater baseline-adjusted improvements in key biochemical parameters, most notably in insulin resistance (fasting insulin and HOMA-IR) and systemic inflammation (hs-CRP). If the observed clinical benefits were primarily mediated by general dietary discipline or overall compliance, a comparable or greater response might have been anticipated in the standard diet group. Instead, these findings suggest that the favorable metabolic and anti-inflammatory changes observed in the nutrigenetic group may be attributable to precision gene–nutrient matching rather than differences in adherence levels, supporting the potential biological value of genotype-tailored dietary strategies.

The additional benefit observed in the nutrigenetic diet group may reflect a more precise dietary alignment with individual genetic requirements for antioxidant enzyme cofactors and substrates. This response could potentially be mediated by variants in genes such as *SIRT1*, whose activity modulates antioxidant gene expression through *FOXO3*-dependent pathways and is sensitive to redox regulation and *APOA2*, whose variants may differentially modulate lipid metabolism and oxidative risk in response to dietary fat composition [67,68,69]. However, without direct measures of tissue gene expression or functional enzyme kinetics, these mechanistic pathways remain hypothetical and require further experimental validation.

To our knowledge, this is one of the few nutrigenetic clinical trials to evaluate between-group differences in antioxidant enzyme activity alongside direct biomarkers of oxidative damage. Most intervention studies addressing oxidative biomarkers in obesity have focused on conventional caloric restriction or antioxidant-rich dietary patterns rather than genotype-guided prescriptions. Previous caloric restriction and Mediterranean diet trials have reported improvements in TAC and SOD activity in individuals with obesity [70,71]. However, concurrent improvements in catalase, GPx, LPO, and 8-isoprostane have been less consistently documented. This variability may partly reflect the use of standardized dietary approaches that do not account for interindividual differences in nutrient metabolism, fatty acid utilization, and nutrigenomic responses [72,73]. Our findings provide evidence suggesting that integrating genetic information into dietary design may contribute to a more robust and antioxidant response.

Clinical trials conducted to date suggest that nutrigenetic interventions can improve dietary adherence, weight management, and specific metabolic outcomes in adults compared with conventional dietary recommendations. However, results across the literature remain heterogeneous due to variations in study design, sample size, intervention duration, and the specific genetic panels used. Current evidence suggests that genotype-based dietary strategies may be particularly relevant for adults with obesity or increased cardiometabolic risk, especially when integrated with baseline phenotypic and biochemical information [36,48,49,74].

To date, relatively few randomized clinical trials from Greece, Canada, and the Netherlands, followed by larger initiatives such as Food4Me and more recent interventions in Mexico, have evaluated nutrigenetic dietary strategies in adults with obesity and insulin resistance. This evidence is even more limited in Latin American populations, highlighting the relevance of the present study. Although the superiority of genotype-guided diets over conventional approaches across different populations remains under investigation, our findings contribute evidence supporting their potential clinical utility [37,49,50,75]. Building on these findings, future research should explore the implementation of personalized nutrigenetic approaches in patients with established cardiovascular disease. Investigating such interventions beyond primary prevention will be important for evaluating their therapeutic potential in reducing cardiometabolic comorbidities, slowing disease progression, and improving patients’ long-term quality of life.

## 5. Limitations

Although the present study provides encouraging evidence supporting the integration of a multi-variant nutrigenetic profile into personalized dietary interventions, some limitations must be acknowledged. While the cohort size met the a priori statistical power requirements for the primary outcome, the sample size remained relatively modest. This reflects the high logistical complexity of human nutrigenetic trials, including strict follow-up protocols, multi-marker genotyping, and specialized biochemical determinations. Future multicenter studies with larger sample sizes are warranted to enable robust genotype-stratified subgroup analyses and validate these findings.

The 8-week intervention period focused primarily on evaluating short-term treatment effects and initial metabolic adaptations. Extended clinical trials with longer follow-up durations are required to assess long-term dietary adherence, weight maintenance, and the durability of the observed cardiometabolic and antioxidant improvements. Moreover, body composition was evaluated using multifrequency bioelectrical impedance analysis (InBody 370) rather than gold-standard methods such as dual-energy X-ray absorptiometry (DXA).

Furthermore, given the extensive panel of secondary outcomes evaluated, baseline-adjusted secondary endpoints with FDR-adjusted *p*-values close to 0.05 should be interpreted with caution as exploratory or supportive evidence, considering potential type I error inflation resulting from multiple testing. Moreover, sex-stratified findings must be viewed as exploratory and hypothesis-generating. Future well-powered precision nutrition trials are needed to determine whether genetic matching exerts true sex-specific effects on cardiometabolic outcomes.

Finally, dietary intake and adherence were monitored using self-reported 24 h dietary recalls and subjective 10-point compliance scales, which inherently carry a risk of recall bias and underreporting. However, dietary adequacy percentages for energy and macronutrients remained close to 100% across groups, and self-reported compliance averaged ~80% throughout the study. The significant improvements observed in objective anthropometric, metabolic, and inflammatory biomarkers provide additional evidence of intervention effects, although they do not independently confirm dietary adherence.

## 6. Future Perspectives

Building upon the metabolic and inflammatory findings observed in this trial, future research will focus on integrating multiomics and molecular approaches to further elucidate the biological mechanisms underlying genotype-guided dietary interventions. Specifically, upcoming trials will incorporate peripheral blood mononuclear cell (PBMC) isolation and adipose tissue biopsies to measure the expression of key target genes and the abundance and activation status of their corresponding proteins, including SIRT1, FOXO3, NF-κB (p65), JNK, and eNOS, using RT-qPCR and Western blotting, respectively, alongside intracellular NAD^+^/NADH ratio quantification, to clarify cellular redox and inflammatory signaling pathways. Furthermore, expanding to larger multicenter cohorts will enable robust genotype-stratified subgroup analyses and single-variant gene-diet interaction models for key loci such as *FTO* (rs9939609), *SIRT1* (rs2273773), and *APOA2* (rs5082), thereby elucidating the specific contribution of individual single-nucleotide variants within the multivariant algorithm. To complement dietary recalls and provide objective biomarkers of dietary adherence, plasma lipidomics using GC-MS/LC-MS should be conducted to track in vivo changes in circulating fatty acid profiles. Finally, integrating 16S rRNA/metagenomic sequencing with fecal short-chain fatty acid quantification will allow us to evaluate the functional role of the gut microbiome-metabolite axis in mediating the metabolic and antioxidant improvements achieved through precision nutrition.

## 7. Conclusions

In summary, an 8-week nutrigenetic dietary intervention tailored to 21 genetic variants associated with lipid metabolism and cardiometabolic risk was associated with greater baseline-adjusted improvements in fasting insulin, estimated insulin resistance (HOMA-IR), and systemic inflammation (hs-CRP), alongside a greater increase in antioxidant enzyme activity (specifically Δ catalase), although the latter did not remain statistically significant after FDR correction, compared with a standard diet in adults with abdominal obesity. While both dietary strategies effectively promoted overall weight loss and fat mass reduction through caloric restriction, the superior metabolic and anti-inflammatory effects observed in the nutrigenetic group highlight the potential of gene-guided nutrition to optimize cardiometabolic health beyond conventional caloric restriction alone.

These findings suggest that precision nutrition targeting individual genetic variability across lipid, glycemic, inflammatory, and redox pathways offers a promising strategy for personalized obesity management. Nevertheless, these results should be interpreted within the context of the study limitations, including the per-protocol analytical framework and statistical power constraints within sex subgroups. Larger, multicenter randomized controlled trials with extended follow-up durations are warranted to confirm the long-term durability, clinical applicability, and generalizability of these nutrigenetic interventions.

## Figures and Tables

**Table 1:** Clinical characteristics of the study participants.

Variables	Total Subjects *n* = 58 % (*n*)	Standard Diet Group *n* = 28 % (*n*)	Nutrigenetic Diet Group *n* = 30 % (*n*)	*p*
Sex				
W	72.4 (42)	75.9 (21)	68.8 (21)	0.667 ^a^
M	27.5 (16)	24.1 (7)	31.3 (9)	
Alcohol consumption				
Yes	68.9 (40)	71.4 (20)	66.6 (20)	0.567 ^a^
No	31.1 (18)	28.6 (8)	33.3 (10)	
Tobacco consumption				
Yes	10.3 (6)	10.8 (3)	10 (3)	0.881 ^b^
No	89.6 (52)	89.2 (25)	90 (27)	
Phsycal activity				
Low	58.6 (34)	62.1 (17)	53.1 (17)	0.829 ^a^
Moderate	20.6 (12)	20.7 (5)	25 (7)	
High	20.6 (12)	17.2 (6)	21.9 (6)	

W: women; M: men. Data are presented as frequency and percentage. ^a^ *p*-values were obtained using the chi-square test. ^b^ *p*-values were obtained using Fisher’s exact test.

**Table 2:** Baseline anthropometric and biochemical variables in the study participants and by study groups.

Variables	Recommendation	Total Subjects *n* = 58	Standard Diet Group *n* = 28	Nutrigenetic Diet Group *n* = 30	*p*
**Anthropometric**					
BMI (kg/m^2^)	18.5–24.9	32.1 ± 5.0	32.0 ± 5.1	32.2 ± 5.2	0.922
BF (%)					
W	<35	44.2 ± 5.2	44.3 ± 5.6	44.2 ± 5.3	0.942
M	<25	38.1 ± 6.3	36.9 ± 5.4	39.4 ± 7.3	0.466
WC (cm)					
W	<88	96.2 ± 8.7	95.0 ± 7.7	97.4 ± 9.7	0.398
M	<102	109.2 ± 13.7	109.0 ± 8.6	109.5 ± 16.8	0.937
**Biochemical**					
Glucose (mg/dL)	<100	104.7 ± 11.5	106.7 ± 11.4	102.7 ± 11.9	0.253
TC (mg/dL)	<200	206.9 ± 36.6	210.8 ± 33.6	203.0 ± 40.6	0.402
TG (mg/dL)	<150	163.4 ± 88.0	166.4 ± 100	160.5 ± 79.2	0.754
HDL-c (mg/dL)					
W	≥50	46.8 ± 11.7	45.5 ± 11.4	48.1 ± 12.7	0.433
M	≥40	45.5 ± 11.8	45.4 ± 10.6	45.7 ± 13.7	0.957
LDL-c (mg/dL)	<100	132.8 ± 31.7	142.7 ± 56.9	122.9 ± 34.6	0.188
VLDL-c (mg/dL)	<30	32.6 ± 14.0	33.2 ± 20	32.1 ± 15.5	0.672
CRI- I (TC/HDL-c)					
W	<4	4.45 ± 1.2	4.71 ± 1.4	4.19 ± 1.3	0.285
M	<5	5.38 ± 1.4	5.52 ± 1.7	5.24 ± 1.1	0.346
CRI-II (LDL-c/HDL-c)					
W	<2	2.74 ± 1.4	2.94 ± 0.8	2.55 ± 1.1	0.158
M	<2.5	3.28 ± 0.5	3.44 ± 0.6	3.12 ± 0.7	0.318
TG/HDL-c ratio					
W	<3	3.53 ± 2.4	3.61 ± 2.4	3.46 ± 2.5	0.844
M	<3.75	4.80 ± 5.0	5.99 ± 7.1	3.61 ± 2.9	0.377
TyG index	<4.68	4.81 ± 0.25	4.83 ± 0.2	4.79 ± 0.2	0.668
Insulin (IU/mL)	5–25	20.6 ± 15.7	20.3 ± 16.8	20.9 ± 15.3	0.882
HOMA-IR	<2.5	5.50 ± 4.6	5.56 ± 5.2	5.44 ± 4.2	0.732

BMI: body mass index; BF (%): body fat percentage; WC: waist circumference; kg: kilograms; cm: centimeters; TC: total cholesterol; TG: triglycerides; HDL-c: high-density lipoprotein cholesterol; LDL-c: low-density lipoprotein cholesterol; VLDL-c: very-low-density lipoprotein cholesterol; CRI- I: Castelli Risk Index I; CRI- II: Castelli Risk Index II; TyG index: triglyceride-glucose index; HOMA-IR: Homeostasis Model Assessment of Insulin Resistance; W: women; M: men. Data are presented as mean ± SD. Comparisons between groups (standard vs. nutrigenetic diet) were performed using Student’s *t*-test or the Mann–Whitney U test, according to the distribution of the data.

**Table 3:** Baseline dietary variables in the study groups.

Variables	Recommendation	Standard Diet Group *n* = 28	Nutrigenetic Diet Group *n* = 30	*p*
Energy (kcal/day)	-	1885 ± 773	1960 ± 843	0.394
CH (%)	55	42.3 ± 11.5	43.3 ± 11.7	0.741
Ps (%)	15	20.3 ± 6.3	19.1 ± 5.6	0.449
TFA (%)	<30	37.3 ± 10.4	37.5 ± 11.8	0.817
SFA (%)	<7	13.0 ± 5.3	11.5 ± 5.3	0.275
MUFA (%)	>10	13.6 ± 4.7	14.7 ± 7.1	0.885
PUFA (%)	10	6.25 ± 3.3	7.58 ± 3.9	0.145
*n*-6:*n*-3 ratio	<5:1	8.29 ± 4.3	7.96 ± 4.2	0.931
Cholesterol (mg)	<300	401.8 ± 219	352.8 ± 229	0.398
Vitamin C (mg)	75	106.7 ± 122	95.7 ± 70.4	0.667
Vitamin D (IU)	600	124.0 ± 82.7	163.8 ± 161.8	0.239
Vitamin E (mg)	15	8.11 ± 7.0	9.12 ± 7.4	0.589
Total fiber (g)	25–35	23.3 ± 16.6	23.3 ± 10.0	0.378
Sodium (mg)	<2000	2065 ± 1509	1865 ± 1058	0.729

kcal: kilocalories; CH: carbohydrates; Ps: protein; TFA: total fatty acids; SFA: saturated fatty acids; MUFA: monounsaturated fatty acids; PUFA: polyunsaturated fatty acids; *n*-6:*n*-3: omega-6:omega-3; %: percentage; g: grams; mg: milligrams; µg: micrograms. Data are presented as mean ± SD. Comparisons between groups (standard vs. nutrigenetic diet) were performed using Student’s *t*-test or Mann–Whitney U test, according to the distribution of the data.

**Table 4:** Changes in anthropometric variables in the study groups.

	Standard Diet Group				Nutrigenetic Diet Group						
Variables	Baseline *n* = 28	4 Weeks *n* = 28	8 Weeks *n* = 28	Δ *n* = 28	Baseline *n* = 30	4 Weeks *n* = 20	8 Weeks *n* = 30	Δ *n* = 30	*p* ^a^	*p* ^b^	*p* ^Δ^
**Anthropometric**											
Weight (kg)	88.2 ± 16.1	86.9 ± 16.5	86.3 ± 16.2	−1.88 ± 2.0	88.9 ± 17.1	86.7 ± 17.0	85.8 ± 16.4	−3.11 ± 2.5	0.004	<0.001	0.045
SMM (kg)	27.9 ± 6.1	27.9 ± 6.2	27.8 ± 7.3	0.01 ± 1.2	28.0 ± 6.2	28.3 ± 6.4	27.7 ± 6.2	−0.26 ± 1.0	0.932	0.214	0.368
BF (kg)	37.6 ± 10.2	36.6 ± 10.5	35.9 ± 10.0	−1.69 ± 1.8	38.2 ± 10.6	36.3 ± 10.9	35.5 ± 10.6	−2.62 ± 2.2	0.005	<0.001	0.085
W	37.8 ± 10.6	36.8 ± 10.9	36.4 ± 11.3	−1.35 ± 1.5	37.3 ± 9.4	35.4 ± 9.7	34.7 ± 9.7	−2.60 ± 2.5	0.016	0.007	0.056
M	37.1 ± 9.9	36.1 ± 9.0	34.5 ± 10.0	−2.65 ± 2.4	40.3 ± 13.4	38.0 ± 14.2	37.6 ± 13.3	−2.68 ± 1.5	0.005	<0.001	0.975
BF (%)								-			
W	44.3 ± 5.6	43.7± 6.5	43.0 ± 7.0	−1.25 ± 1.4	44.2 ± 5.3	43.6 ± 5.9	41.61 ± 5.0	−2.59 ± 2.5	0.265	0.017	0.056
M	36.9 ± 5.4	36.5 ± 4.9	35.0 ± 6.7	−1.94 ± 2.4	39.4 ± 7.3	37.8 ± 7.8	37.7 ± 7.6	−1.70 ± 1.5	0.056	0.030	0.796
WC (cm)											
W	95.0 ± 7.7	91.4 ± 7.5	90.1 ± 8.5	−4.85 ± 3.0	97.4 ± 9.7	93.6 ± 9.1	91.7 ± 10.1	−5.70 ± 3.1	<0.001	0.004	0.391
M	109.0 ± 8.6	105.7 ± 7.9	103.3 ± 11.0	−5.65 ± 2.1	109.5 ± 16.7	105.1 ± 17.3	103.3 ± 17.0	−6.20 ± 2.1	0.003	<0.001	0.626
BMI (kg/m^2^)	32.0 ± 5.1	31.7 ± 5.0	31.4 ± 5.1	−0.61 ± 0.6	32.2 ± 5.2	31.8 ± 5.6	31.1 ± 5.5	−1.03 ± 1.1	0.626	0.004	0.344

SMM: skeletal muscle mass; BF: body fat; WC: waist circumference; BMI: body mass index; kg: kilograms; %: percentage; cm: centimeters; Δ: change from baseline to final measurement. Data are presented as mean ± SD. *p* ^a^: intragroup comparisons between baseline and final measurements within the standard diet group; *p* ^b^: intragroup comparisons between baseline and final measurements within the nutrigenetic diet group; *p* ^Δ^: comparison of changes (Δ) between the standard and nutrigenetic diet groups. Comparisons were performed using Student’s *t*-test or the Mann–Whitney U test, according to the distribution of the data.

**Table 5:** Changes in biochemical variables in the study groups.

	Standard Diet Group				Nutrigenetic Diet Group						
Variables	Baseline *n* = 28	4 Weeks *n* = 28	8 Weeks *n* = 28	Δ *n* = 28	Baseline *n* = 30	4 Weeks *n* = 30	8 Weeks *n* = 30	Δ *n* = 30	*p* ^a^	*p* ^b^	*p* ^Δ^
Glucose (mg/dL)	106.7 ± 11.4	104.3 ± 9.6	103.6 ± 10.0	−3.10 ± 10.7	102.7 ± 11.9	101.4 ± 11.1	100.9 ± 8.9	−1.80 ± 8.2	0.711	0.069	0.542
TC (mg/dL)	210.8 ± 34.2	204.6 ± 42.1	208.1 ± 44.1	−2.61 ± 34.0	203.0 ± 40.6	198.2 ± 44.2	195.8 ± 42.4	−7.17 ± 22.5	0.257	0.235	0.773
TG (mg/dL)	166.4 ± 100	160.9 ± 105	151.1 ± 95	−15.3 ± 51.7	160.5 ± 79.2	150.1 ± 85.2	140.9 ± 63.1	−19.6 ± 42.6	0.374	0.235	0.749
HDL-c (mg/dL)											
W	45.5 ± 11.4	44.3 ± 13.7	43.6 ± 10.6	−1.86 ± 7.1	48.1 ± 12.7	47.9 ± 9.9	48.1 ± 11.9	0.05 ± 7.6	0.265	0.812	0.771
M	45.4 ± 10.6	46.0 ± 11	46.6 ± 8.2	1.29 ± 7.2	45.7 ± 13.7	43.5 ± 7.7	42.3 ± 9.6	−3.33 ± 5.2	0.757	0.665	0.159
LDL-c (mg/dL)	142.7 ± 56.9	135.8 ± 33.2	131.9 ± 36.8	−10.8 ± 60.2	122.9 ± 34.6	119.2 ± 38.0	116.5 ± 37.4	−6.33 ± 20.2	0.193	0.892	0.500
VLDL-c (mg/dL)	33.2 ± 20.0	32.8 ± 21.1	30.1 ± 19.0	−3.07 ± 10.3	32.1 ± 15.5	31.0 ± 17.0	28.1 ± 12.6	−3.92 ± 8.5	0.370	0.321	0.731
TG/HDL-c ratio											
W	3.77 ± 3.7	3.80 ± 3.75	3.87 ± 3.7	0.10 ± 2.6	3.63 ± 2.5	3.38 ± 2.5	3.27 ± 1.4	−0.36 ± 0.9	0.213	0.540	0.461
M	5.37 ± 5.8	4.73 ± 5.8	4.48 ± 6.4	−0.89 ± 1.8	5.56 ± 3.2	5.58 ± 3.2	5.66 ± 2.4	0.11 ± 1.0	0.954	0.430	0.191
CRI-I (TC/HDL-c)											
W	4.71 ± 1.4	4.58 ± 1.4	4.61 ± 1.4	−0.10 ± 0.9	4.19 ± 1.3	4.15 ± 1.3	4.13 ± 1.3	−0.06 ± 0.6	0.653	0.580	0.771
M	5.52 ± 1.7	5.59 ± 1.8	5.73 ± 1.6	0.21 ± 1.0	5.24 ± 1.1	5.27 ± 1.1	5.32 ± 1.5	0.08 ± 0.5	0.321	0.770	0.748
CRI-II (LDL-c/HDL-c)											
W	2.94 ± 0.8	2.76 ± 0.8	2.63 ± 0.8	−0.31 ± 0.9	2.55 ± 1.1	2.54 ± 1.1	2.55 ± 1.1	−0.00 ± 0.1	0.118	0.509	0.221
M	3.44 ± 0.6	3.34 ± 0.6	3.13 ± 0.7	0.34 ± 0.9	3.12 ± 0.7	3.10 ± 0.7	3.09 ± 1.0	−0.03 ± 0.5	0.213	0.910	0.339
TyG index	4.83 ± 0.2	4.79 ± 0.2	4.77 ± 0.2	−0.06 ± 0.1	4.79 ± 0.2	4.77 ± 0.2	4.75 ± 0.2	−0.04 ± 0.1	0.542	0.965	0.932
Insulin (μIU/mL)	20.3 ± 16.8	17.2 ± 16.2	16.1 ± 11.0	−4.14 ± 8.5	20.9 ± 15.3	15.2 ± 10.8	12.4 ± 7.8	−8.41 ± 8.7	0.064	<0.001	0.072
HOMA-IR	5.56 ± 5.2	4.71 ± 5.2	4.31 ± 3.4	−1.25 ± 2.4	5.44 ± 4.2	4.06 ± 2.7	3.32 ± 2.1	−2.12 ± 2.4	0.542	0.004	0.181
hs-CRP (mg/L)	9.61 ± 14.0	7.61 ± 9.6	6.67 ± 5.6	−2.94 ± 9.92	8.11 ± 7.7	5.71 ± 7.7	3.77 ± 2.1	−4.34 ± 6.8	0.121	0.001	0.615

TC: total cholesterol; TG: triglycerides; HDL-c: high-density lipoprotein cholesterol; LDL-c: low-density lipoprotein cholesterol; VLDL-c: very-low-density lipoprotein cholesterol; CRI-I: Castelli Risk Index I; CRI-II: Castelli Risk Index II; TyG index: triglyceride-glucose index; HOMA-IR: Homeostasis Model Assessment of Insulin Resistance; hs-CRP: high-sensitivity *C*-reactive protein; Δ: change from baseline to final measurement. Data are presented as mean ± SD. *p* ^a^: intragroup comparisons between baseline and final measurements within the standard diet group; *p* ^b^: intragroup comparisons between baseline and final measurements within the nutrigenetic diet group; *p* ^Δ^: comparison of changes (Δ) between the standard and nutrigenetic diet groups. Comparisons were performed using Student’s *t*-test or the Mann–Whitney U test, according to the distribution of the data.

**Table 6:** Changes in the prevalence of cardiometabolic disorders in the study groups.

	Standard Diet Group ^a^		Nutrigenetic Diet Group ^b^				
Variable	Baseline *n* = 28 % (*n*)	Final *n* = 28 % (*n*)	Baseline *n* = 30 % (*n*)	Final *n* = 30 % (*n*)	*p* ^a^	*p* ^b^	*p* ^Δ^
Hypercholesterolemia	64.3 (18)	50 (14)	43.3 (13)	33.3 (10)	0.289	0.375	0.114
Hypoalphalipoproteinemia	50 (14)	64.3 (18)	53.3 (16)	63.3 (19)	0.219	0.375	0.638
Elevated LDL-c	85.7 (24)	75 (21)	76.7 (23)	56.7 (17)	0.453	0.031	0.547
Hypertriglyceridemia	46.4 (13)	35.7 (10)	46.7 (14)	36.7 (11)	0.508	0.453	0.803
Hyperglycemia	71.4 (20)	64.3 (18)	53.3 (16)	50 (15)	0.687	1.000	0.481
TG/HDL-c index	42.9 (12)	46.4 (13)	46.7 (14)	36.7 (11)	1.000	0.375	0.451
CRI-I (TC/HDL-c)	64.3 (18)	64.3 (18)	50 (15)	43.3 (13)	1.000	0.687	0.554
CRI-II (LDL-c/HDL-c)	82.1 (23)	89.3 (25)	76.7 (23)	63.3 (19)	0.375	0.375	0.718
TyG index	67.9 (19)	57.1 (16)	73.3 (22)	53.3 (16)	0.453	0.031	0.068
HOMA-IR	82.1 (23)	71.4 (20)	83.3 (25)	50 (15)	0.250	0.002	0.114

LDL-c: low-density lipoprotein cholesterol; TG: triglycerides; HDL-c: high-density lipoprotein cholesterol; TC: total cholesterol; CRI-I: Castelli Risk Index I; CRI- II: Castelli Risk Index II; TyG index: triglyceride-glucose index; HOMA IR: Homeostasis Model Assessment of Insulin Resistance. *p* ^a^: comparison between baseline and final prevalence within the standard diet group; *p* ^b^: comparison between baseline and final prevalence within the nutrigenetic diet group; *p* ^Δ^: comparison of changes between groups.

**Table 7:** Changes in antioxidant and oxidative damage biomarkers in the study groups.

	Standard Diet Group ^a^			Nutrigenetic Diet Group ^b^					
Variables	Baseline *n* = 28	Final *n* = 28	Δ *n* = 28	Baseline *n* = 30	Final *n* = 30	Δ *n* = 30	*p* ^a^	*p* ^b^	*p* ^Δ^
**Antioxidant biomarkers**									
Catalase (U/mL)	16.0 ± 10.0	18.6 ± 10.7	2.65 ± 1.63	16.9 ± 12.8	22.1 ± 16.3	5.16 ± 6.4	<0.001	<0.001	0.040
GPx (U/mL)	0.54 ± 0.34	0.58 ± 0.12	0.04 ± 0.23	0.45 ± 0.11	0.59 ± 0.11	0.14 ± 0.1	0.291	<0.001	0.043
SOD (U/mL)	0.06 ± 0.01	0.07 ± 0.01	0.01 ± 0.01	0.06 ± 0.01	0.07 ± 0.01	0.01 ± 0.0	<0.001	<0.001	0.464
TAC (μM/CRE)	384.1 ± 68	426.9 ± 73	42.82 ± 84	370.6 ± 55	411.2 ± 73	40.57 ± 64	0.010	0.001	0.906
**Oxidative damage biomarkers**									
LPO (µM)	2.21 ± 2.26	1.31 ± 1.09	−0.89 ± 2.4	2.80 ± 3.00	1.35 ± 1.02	−1.44 ± 3.1	0.063	0.015	0.463
8-isoprostane (pg/mL)	29.9 ± 3.42	27.1 ± 3.16	−2.79 ± 4.1	29.4 ± 3.66	26.8 ± 2.32	−2.55 ±4.9	0.001	0.007	0.841
oxLDL (ng/mL)	610 ± 710	534.6 ± 412	−75.6 ± 470	1142 ± 1412	1063 ± 1239	−78.3 ± 313	0.386	0.167	0.979
oxHDL (ng/mL)	5.33 ± 2.4	5.10 ± 1.8	−0.22 ± 1.6	5.75 ± 2.9	5.47 ± 2.7	−0.28 ± 0.9	0.454	0.103	0.865

GPx: glutathione peroxidase; SOD: superoxide dismutase; TAC: total antioxidant capacity; LPO: lipid peroxidation; oxLDL: oxidized low-density lipoprotein; oxHDL: oxidized high-density lipoprotein; Δ: change from baseline to final measurement. Data are presented as mean ± SD. *p* ^a^: intragroup comparisons between baseline and final measurements within the standard diet group; *p* ^b^: intragroup comparisons between baseline and final measurements within the nutrigenetic diet group; *p* ^Δ^: comparison of changes (Δ) between the standard and nutrigenetic diet groups. Comparisons were performed using Student’s *t*-test or the Mann–Whitney U test, according to the distribution of the data.

**Table 8:** Univariate analysis of covariance (ANCOVA) models adjusted for baseline values, age, and sex, with correction for multiple testing.

Outcome Variable	Control Group (*n* = 28) Adjusted Mean (95% CI)	Nutrigenetic Group (*n* = 30) Adjusted Mean (95% CI)	Between-Group Adjusted Difference (95% CI)	ANCOVA *p*-Value	Multiplicity Adjusted *p*-Value (FDR)
**Primary outcome**					
Body fat mass (kg)	37.2 (36.1–38.3)	36.4 (35.3–37.4)	−0.83 (−2.37–0.71)	0.285	
**Secondary outcome**					
Body weight (kg)	87.4 (86.4–88.3)	86.5 (85.6–87.4)	−0.89 (−2.16–0.38)	0.167	0.301
BF (%)	42.0 (40.9–43.2)	41.6 (40.5–42.7)	−0.42 (−2.04–1.18)	0.596	0.671
WC (cm)	95.7 (94.4–97.0)	94.6 (93.3–95.9)	−1.12 (−2.98–0.74)	0.233	0.350
Insulin (μIU/mL)	16.7 (15.1–18.2)	12.8 (11.3–14.3)	−3.86 (−6.00–−1.73)	0.001	0.005
HOMA-IR	4.37 (3.93–4.81)	3.33 (2.90–3.76)	−1.03 (−1.65–−0.42)	0.001	0.005
hs-CRP (mg/L)	6.11 (4.98–7.24)	3.82 (2.75–4.89)	−2.29 (−3.85–−0.73)	0.005	0.015
Catalase (U/mL)	19.2 (17.6–20.9)	21.6 (20.0–23.2)	2.38 (0.07–4.70)	0.043	0.097
SOD	0.07 (0.07–0.08)	0.07 (0.07–0.08)	−0.002 (−0.011–0.006)	0.555	0.671
GPx (U/mL)	0.57 (0.54–0.60)	0.61 (0.58–0.64)	0.03 (−0.00–0.07)	0.112	0.252

BF: body fat; WC: waist circumference; HOMA-IR: Homeostasis Model Assessment of Insulin Resistance; hs-CRP: high-sensitivity *C*-reactive protein; SOD: superoxide dismutase; GPx: glutathione peroxidase. Values are adjusted means (estimated marginal means, EMMs) derived from analysis of covariance (ANCOVA) models at week 8, using baseline values, age, and sex as covariates and treatment group as a fixed factor. The “Between-Group Adjusted Difference” is calculated as EMM Nutrigenetic—EMM Standard and reported with corresponding 95% confidence intervals (95% CIs). Multiplicity across secondary endpoints (m = 9) was controlled using the Benjamini–Hochberg false discovery rate (FDR) procedure. Both unadjusted *p*-values and FDR-adjusted *p*-values are presented; statistical significance was set at an FDR-adjusted q-value < 0.05.

## Data Availability

The original contributions presented in this study are included in the article and its Supplementary Materials.

## Supplementary Materials

The following supporting information can be downloaded at: https://www.mdpi.com/article/10.3390/nu18213519/s1, Table S1: Macronutrients and micronutrients composition of the standard and nutrigenetic diets; Table S2: Cut-off values used to define cardiometabolic abnormalities; Table S3: Genotype and allele frequencies in the nutrigenetic diet group; Table S4: Dietary intake adequacy by intervention group; Table S5: Self-reported dietary adherence by intervention group.

## Abbreviations

The following abbreviations are used in this manuscript:

ABCA1	ATP-binding cassette transporter A1
APOA1	Apolipoprotein A1
APOA2	Apolipoprotein A2
APOA5	Apolipoprotein A5
APOB	Apolipoprotein B
APOC3	Apolipoprotein *C*-III
APOE	Apolipoprotein E
BMI	Body mass index
CETP	Cholesteryl ester transfer protein
CVD	Cardiovascular disease
CDKN2B-AS1	Antisense non-coding RNA in the INK4 locus
CD36	Cluster of differentiation 36
CH	Carbohydrate
CI	Confidence interval
DHA	Docosahexaenoic acid
DNA	Deoxyribonucleic acid
eNOS	Endothelial nitric oxide synthase
EPA	Eicosapentaenoic acid
FABP2	Fatty acid-binding protein 2
FTO	Alpha-ketoglutarate-dependent dioxygenase FTO
GPx	Glutathione peroxidase
HDL-c	High-density lipoprotein cholesterol
hs-CRP	High-sensitivity *C*-reactive protein
IL-6	Interleukin 6
LCAT	Lecithin–cholesterol acyltransferase
LDL-c	Low-density lipoprotein cholesterol
LIPC	Hepatic lipase
LPL	Lipoprotein lipase
MTHFR	Methylenetetrahydrofolate reductase
MTTP	Microsomal triglyceride transfer protein
MUFA	Monounsaturated fatty acids
NF-κB	Nuclear factor kappa B
NO	Nitric oxide
*n*-3	Omega-3
*n*-6	Omega-6
AO	Abdominal obesity
PPARα	Peroxisome proliferator-activated receptor alpha
PON1	Paraoxonase 1
PUFA	Polyunsaturated fatty acids
ROS	Reactive oxygen species
SD	Standard deviation
SFA	Saturated fatty acids
SIRT1	Sirtuin 1
SOD	Superoxide dismutase
TAC	Total antioxidant capacity
T2DM	Type 2 diabetes mellitus
TC	Total cholesterol
TFA	Total fatty acids
TG	Triglycerides
Trans FA	Trans fatty acids
VLDL-c	Very-low-density lipoprotein cholesterol
WC	Waist circumference
ZPR1	Zinc finger protein ZPR1
