# Supplementary material for: Effects of a Nutrigenetic Diet on Fat Mass Loss, Insulin Resistance, and Subclinical Inflammation in Adults with Abdominal Obesity: A Randomized Controlled Trial—The INNUPREC Study
Source: Nutrients. 2026 Oct 26;18(21):3519. doi: 10.3390/nu18213519 (PMC13764880; doi:10.3390/nu18213519)
Supplement: Supplementary file 1 [file nutrients-18-03519-s001.zip › nutrients-4589123-supplementary.pdf]

Table S1. Macronutrients and micronutrients composition of the standard and nutrigenetic diets.

Nutrient	Standard diet group	Nutrigenetic diet group	
		Pattern 1	Pattern 2
CH (%)	55	55	35
Ps (%)	20	25	25
TFA (%)	25	20	40
SFA (%)	5	5	7
MUFAs (%)	12	7	20
PUFA (%)	8	8	13
Cholesterol (mg)	<300	<300	<300
Vitamin E (mg)	15	15	15
Vitamin C (mg)	75	75	75
Vitamin D (UI)	600	600	600
Polyphenols (mg)	1000	1000	1000

CH: carbohydrates; Ps: protein; TFA: total fatty acids; SFA: saturated fatty acids; MUFA: monounsaturated fatty acids; PUFA: polyunsaturated fatty acids; %: percentage; IU: international units; mg: milligrams; μg: micrograms

Table S2. Cut-off values used to define cardiometabolic abnormalities.

Index	Cut-off value
Hypercholesterolemia	≥ 200 mg/dL
Hypertriglyceridemia	≥ 150 mg/dL
Hypoalphalipoproteinemia	W: <5 0 mg/dL; M: < 40 mg/dL
Elevated LDL-c	≥ 100 mg/dL
TG/HDL-c ratio	W: < 3; M: < 3.75
CRI-I (TC/HDL-c)	W: < 5; M: <4
CRI-II (LDL-c/HDL-c)	W: < 2; M: < 2.5
HOMA-IR	≥ 2.5
TyG index	≥ 4.68

TC: total cholesterol; TG: triglycerides; HDL-c: high-density lipoprotein cholesterol; LDL-c: low-density lipoprotein cholesterol: CRI-I: Castelli Risk Index I; CRI-II: Castelli Risk Index II; HOMA-IR: Homeostasis Model Assessment of Insulin Resistance; TyG: triglyceride-glucose index; W: women; M: men.

Table S3. Genotype and allele frequencies in the nutrigenetic diet group.

Gene/variant (rsID)	Referece/risk allele	Allele frequency %	Genotype frequency %
ABCA1 rs2230806	G/A	G: 64.1%<br>A: 35.9%	GG: 46.9%<br>GA: 34.4%<br>AA: 18.7%
APOA1 rs670	G/A	G: 82.8%<br>A: 17.2%	GG: 65.7%<br>GA: 34.3%<br>AA: 0%
APOA2 rs5082	T/C	T: 71.8%<br>C: 28.2%	TT: 50%<br>TC: 43.7%<br>CC: 6.3%
APOA5 rs662799	T/C	T: 82.8<br>C: 17.2	TT: 68.7%<br>TC: 28.2%<br>CC: 3.1%
APOB rs693	C/T	C: 32.8%<br>T: 67.2%	CC: 12.5%<br>CT: 40.6%<br>TT: 46.9%
APOC3 rs5128	C/G	C: 79.6%<br>G: 20.4%	CC: 65.6%<br>CG: 28.1%<br>GG: 6.3%
APOE<br>rs7412<br>rs429358	 C/T<br>T/C	 C: 96.9% T: 3.1%<br>T: 92.2%. C: 7.8%	E3/E3: 87.5%<br>E3/E4: 9.4%<br>E4/E4: 3.1%
CDKN2B-AS1 rs7865618	A/G	A: 81.2%<br>G: 18.8%	AA: 65.6%<br>AG: 31.3%<br>GG: 3.1%
CD36 rs1761667	G/A	G: 57.8%<br>A: 42.2%	GG: 28.1%<br>GA: 59.4%<br>AA: 12.5%
CETP rs708272	G/A	G: 59.3%<br>A: 40.7%	GG: 37.5%<br>GA: 43.7%<br>AA: 18.8
FABP2 rs1799883	G/A	G: 68.7%<br>A: 31.3%	GG: 46.9%<br>GA: 43.7%<br>AA: 9.4%
FTO rs9939609	T/A	T: 32.8%<br>A: 67.2%	TT: 15.6%<br>TA: 34.4%<br>AA: 50%
LIPC rs1800588	C/T	C: 50%<br>T: 50%	CC: 18.7%<br>CT: 62.6%<br>TT: 18.7 %

LPL rs320	G/T	G: 68.7%<br>T: 31.3%	GG: 46.9%<br>GT: 43.7%<br>TT: 9.4%
MTFHR rs1801133	C/T	C: 51.5%<br>T: 48.5%	CC: 18.7%<br>CT: 65.6%<br>TT: 15.7%
MTTP rs1800591	G/T	G: 68.7%<br>T: 31.3%	GG: 50%<br>GT: 37.5%<br>TT: 12.5%
NFKB1 rs28362491	INS/DEL	INS: 39%<br>DEL: 61%	INS/INS: 0%<br>INS/DEL: 78.1%<br>DEL/DEL: 21.9%
PON1 rs854560	A/T	A: 67.2%<br>T: 32.8%	AA: 46.9%<br>AT: 40.6%<br>TT: 12.5%
SIRT1 rs7896005	A/G	A: 43.7%<br>G: 56.3%	AA: 28.1%<br>AG: 31.2%<br>GG: 40.7%
ZPR1 rs964184	C/G	C: 65.6%<br>G: 34.4%	CC: 40.6%<br>CG: 50%<br>GG: 9.4%

Data are presented as percentages.

Table S4. Dietary intake adequacy by intervention group.

Dietary component	Standard diet group n = 28	Nutrigenetic diet group n = 30	p-value
Energy intake (%)	103.7 ± 5.8	103.2 ± 8.0	0.768
Protein intake (%)	107.9 ± 6.4	101.6 ± 6.5	<0.001
Carbohydrate intake (%)	96.0 ± 2.1	99.5 ± 5.2	0.002
Total Fat intake (%)	102. 2 ± 4.7	100.8 ± 4.5	0.223
Saturated Fat intake (%)	110.6 ± 6.4	114.9 ± 8.3	0.029
Monounsaturated fat intake (%)	99.9 ± 6.7	97.1 ± 5.9	0.095
Polyunsaturated fat intake (%)	100. 6 ± 6.4	97.2 ± 7.5	0.069
Cholesterol intake	87.6 ± 34.3	96.2 ± 43.4	0.400
Vitamin C intake	124.6 ± 49.4	109.3 ± 59.6	0.286
Vitamin D intake	80.4 ± 10.8	79.6 ± 9.0	0.758
Vitamin E intake	84.6 ± 15.0	86.8 ± 14.4	0.567

Dietary intake adequacy was calculated as the percentage of reported intake relative to prescribed daily intake for each dietary component. Values are presented as mean ± SEM.

Table S5. Self-reported dietary adherence by intervention group.

	Standard diet group			Nutrigenetic diet group					
**Variable**	Week 4 n=28	Week 8 n=28	Δ n=28	Week 4 n=30	Week 8 n=30	Δ n=30	p [a]	p [b]	p [Δ]
Self-reported Dietary adherence Scale: 1-10	8.20 ± 0.7	8.03 ± 0.8	-0.16 ± 0.8	8.53 ± 0.7	7.95 ± 1.0	-0.57 ± 0.6	0.294	<0.001	**0.032**

Dietary adherence was self-reported using a 10-point scale (1-10). Values are presented as mean ± SD. Δ represents the change from week 4 to week 8. p^a: within-group comparison between weeks 4 and 8 in the standard diet group; p^b: within-group comparison between weeks 4 and 8 in the nutrigenetic diet group; p^{Δ}: between-group comparison of changes (Δ).
